# Towards a validated glossary of usability attributes for the evaluation of wearable robotic devices

**DOI:** 10.1186/s12984-024-01312-1

**Published:** 2024-02-28

**Authors:** Diana Herrera-Valenzuela, Jan T. Meyer, Antonio J. del-Ama, Juan C. Moreno, Roger Gassert, Olivier Lambercy

**Affiliations:** 1 International Doctoral School, Rey Juan Carlos University, Madrid, Spain; 2 Biomechanics and Technical Aids Unit, National Hospital for Paraplegics, Toledo, Spain; 3 Rehabilitation Engineering Laboratory, Department of Health Sciences and Technology, ETH Zurich, Zurich, Switzerland; 4 School of Science and Technology, Department of Applied Mathematics, Materials Science and Engineering and Electronic Technology, Rey Juan Carlos University, Móstoles, Madrid, Spain; 5 Neural Rehabilitation Group, Cajal Institute, CSIC–Spanish National Research Council, Madrid, Spain; 6 Unit of Neurorehabilitation, Biomechanics and Sensorimotor Function (HNP-SESCAM), Associated Unit of R&D&I to the CSIC, Toledo, Spain; 7 Future Health Technologies, Singapore-ETH Centre, Campus for Research Excellence and Technological Enterprise (CREATE), Singapore, Singapore

## Abstract

**Background:**

Despite technical advances in the field of wearable robotic devices (WRD), there is still limited user acceptance of these technologies. While usability often comes as a key factor influencing acceptance, there is a scattered landscape of definitions and scopes for the term. To advance usability evaluation, and to integrate usability features as design requirements during technology development, there is a need for benchmarks and shared terminology. These should be easily accessible and implementable by developers.

**Methods:**

An initial set of usability attributes (UA) was extracted from a literature survey on usability evaluation in WRD. The initial set of attributes was enriched and locally validated with seven developers of WRD through an online survey and a focus group. The locally validated glossary was then externally validated through a globally distributed online survey.

**Results:**

The result is the Robotics Usability Glossary (RUG), a comprehensive glossary of 41 UA validated by 70 WRD developers from 17 countries, ensuring its generalizability. 31 of the UA had high agreement scores among respondents and 27 were considered highly relevant in the field, but only 11 of them had been included as design criteria by the respondents.

**Conclusions:**

Multiple UA ought to be considered for a comprehensive usability assessment. Usability remains inadequately incorporated into device development, indicating a need for increased awareness and end-user perspective. The RUG can be readily accessed through an online platform, the Interactive Usability Toolbox (IUT), developed to provide context-specific outcome measures and usability evaluation methods. Overall, this effort is an important step towards improving and promoting usability evaluation practices within WRD. It has the potential to pave the way for establishing usability evaluation benchmarks that further endorse the acceptance of WRD.

**Supplementary Information:**

The online version contains supplementary material available at 10.1186/s12984-024-01312-1.

## Introduction

Over the last decades, we have witnessed an outstanding evolution in the field of wearable robotic devices (WRD) for rehabilitation and assistance. However, despite technical advances, user acceptance and adoption of these technologies is still very limited [1]. This fact is increasingly attracting the interest of researchers in the WRD field with the aim of better understanding its causes and the limiting factors of the user experience in human–robot interactions [2]. Of particular importance, studies have shown the limited evaluation of user satisfaction with WRD [3], the lack of validated tools to assess devices from the user’s perspective [4], and the need to improve their usability [1].

When it comes to usability, there is a scattered landscape of definitions and scopes for the term. The best-known standard related to usability of human–robot interactions is ISO 9241-11, which defines usability as “the extent to which the user’s physical, cognitive and emotional responses that result from the use of a system, product, or service meet the user’s needs and expectation” [5]. However, only a few WRD studies end up using the exact terminology the standard provides, underlining the difficulty in capturing the complex construct of usability by the means of only three dimensions: effectiveness, efficiency, and satisfaction. As a consequence, other models including further dimensions have been proposed to evaluate usability in assistive technologies [6–9], demonstrating that technology developers more often refer to usability using a broader scope of terms, hereinafter called “usability attributes” (UA). The definition of such UA is often blurry, offering the possibility for different interpretations based on the educational background, the language, as well as application context. Consequently, as of now, there exist no validated definitions of UA that are easily accessible and, more importantly, that were agreed upon by WRD developers. Only once the field establishes an agreement upon specific UA with their respective definitions, can we ensure the WRD community evaluates the same things and provides data that can be more easily compared across devices and studies.

In this regard, open-source benchmarks for the evaluation of WRD have been developed recently in two coordinated European efforts: Eurobench [10] and the European Cooperation in Science and Technology (COST) action for Wearable Robotics [11]. Eurobench aimed to create a framework for applying benchmarking methodology on bipedal robotic systems, including lower limb WRD and robotic humanoids. To run the evaluations proposed in their framework, two facilities with standardized equipment and settings to evaluate lower limb WRD were set up in Europe. Only one of the 75 protocols developed in the Eurobench framework addresses the usability of WRD. This evaluation is conducted through a questionnaire including the attributes acceptability, perceptibility, and functionality. The questionnaire evaluates usability by asking if the device is useful to the user and provides a scoring system based on the three dimensions stated by ISO 9241-11 [12]. Additionally, the protocol is limited only to lower limb WRD, has limited accessibility for developers around the world due to the specialized setups required to evaluate the technologies, and is only applicable to devices in advanced development stages with Technology Readiness Levels (TRL) ≥ 7. On the other hand, the first objective of the COST action for wearable robotics was to create a common understanding of terms and concepts related to wearable robotics among fields of expertise in general. Nevertheless, their vocabulary is not specific to usability or user experience. As such, the term usability itself was not included, but the UA *cognitive load*, *mental fatigue*, *robustness*, and *wearability* were separately considered [11]. This further highlights the need for a more comprehensive, usability-focused framework to define and evaluate the usability of WRD at any TRL.

With a similar motivation, the committee F48 on Exoskeletons and Exosuits formed by the American Society for Testing and Materials (ASTM) has been working to develop voluntary consensus standards for WRD since 2017. They have a subcommittee specifically devoted to defining a Standard Terminology for these WRD, which published the standard F3323-21 with the proposed terms and definitions [13]. Nonetheless, this standard is not related to usability, is not open-access, and was not externally validated, thus having limited accessibility and applicability among WRD developers.

To push usability evaluation and integrate usability features as design requirements during technology development, we need to create benchmarks and shared terminology that can be unequivocally understood, are easily accessible and implementable by WRD researchers and developers. To this end, the Interactive Usability Toolbox (IUT) was developed at ETH Zurich [14]. It takes the form of an online platform aimed at increasing and improving usability evaluation practices during the development of WRD [15]. The Toolbox facilitates the search and selection of context-specific outcome measures and usability research methods, including the option to select specific UA as part of the intended context. To guarantee the comprehensiveness, generalizability and validity of the UA, which are the starting point to recommend specific usability evaluation methods, we aimed to develop an internationally validated glossary of UA as part of the IUT. The objective of this paper is to describe the process of building and externally validating the Robotics Usability Glossary (RUG), a glossary with consensus-based definitions for each commonly used UA. Specifically, we provide the results of a two-step validation consisting first of a local evaluation with usability experts, followed by an online survey administered to developers of WRD around the world to assess the external validity of this glossary. These agreed UA should then become the basis to find and create more widely accepted benchmarks for the usability evaluation of WRD.

## Methods

### Study design

**Fig. 1:**
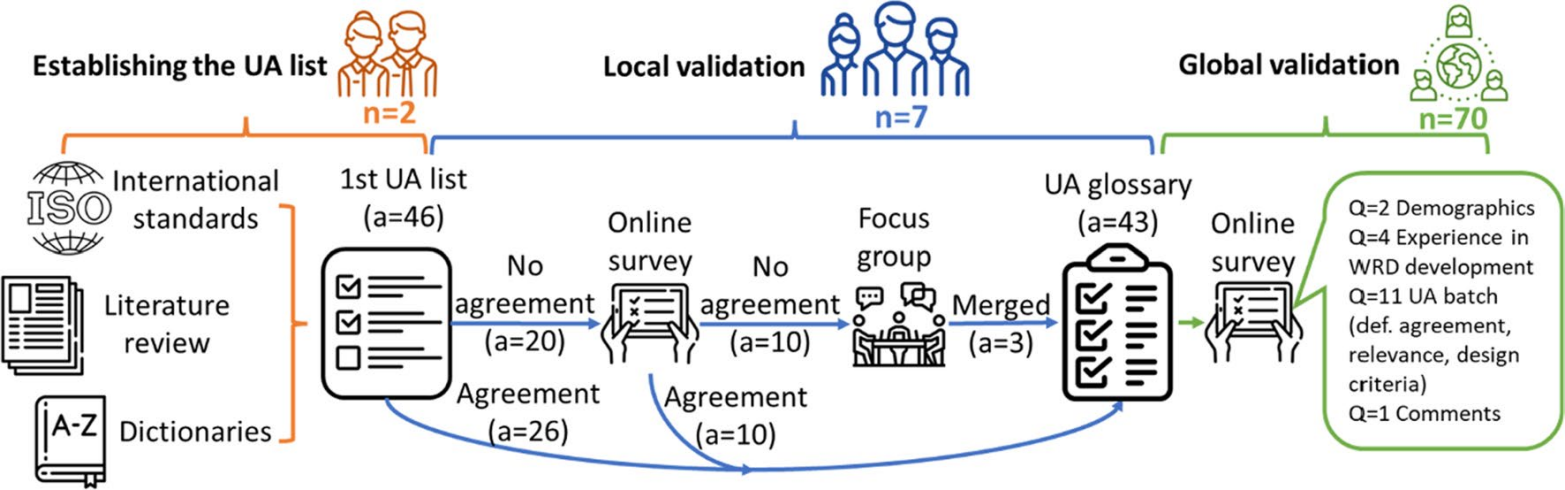
Schematics of the methodology followed to build the UA glossary, validate it locally and launch an online survey to validate it worldwide. The acronyms correspond to the number of developers (n), the number of usability attributes (a), and the number of questions (Q)

An initial set of UA was extracted from a literature survey on usability evaluation in WRD. The initial set of attributes was enriched and locally validated with seven developers of WRD through an online survey and a focus group, leading to a reasonable consensus. The locally validated glossary was then externally validated through a globally distributed online survey. The current study purposely targeted only technology developers because they are mostly the ones conducting and designing usability evaluations or WRD. Therefore, we aimed to reach a consensus among them. Figure 1 summarizes the overall methodology. The details of the process of building the glossary and of the two-step validation are described in the following sections.

#### Establishing the UA list

The first set of UA was gathered based on a literature survey on how usability is assessed in the field of WRD, mostly from other models proposed for usability evaluation [6–9]. The resulting data was summarized in 46 UA that encompass the overall usability of WRD. Previously available definitions were retrieved from their respective papers when available, from standardized guidelines such as ISO 9241-11, from international health organizations like the World Health Organization (WHO) and the Agency for Healthcare Research and Quality (AHRQ), or from English dictionaries (e.g. Cambridge Dictionary, Oxford English Dictionary). The definition that best fit the attribute with respect to WRD was selected, based on the agreement of the two main study coordinators (DHV, JTM).

#### Local validation

UA definitions for which the two study coordinators did not reach a consensus were discussed with a group of seven local WRD developers through an online questionnaire, where the respondents rated with a 5-point Likert scale their agreement with the provided definition(s) of each UA, as well as the applicability of each attribute for the development of WRD. The definitions with average agreement scores of at least 4.0 were thus considered locally validated and not further discussed. The remaining UA were discussed with four of the respondents of the survey during a focus group aimed at (i) improving the definitions based on the available ones and (ii) deciding to potentially merge UA with similar definitions. Despite all seven local developers being invited to participate in the focus group, only 4 of them could participate due to time availability. The session was moderated by the study coordinators (DHV, JTM). All the descriptions built during this session were scored once again by six of the respondents from the initial local survey in a second online survey.

Both surveys were reviewed and tested before being distributed to guarantee the understandability of the questions and face validity of the survey. Comment boxes were always included to gather further insights from the respondents about the definition of each UA. Before starting the study, the research aims and methods were discussed and approved among the authors, assuring that face validity was established.

#### Global validation

With the locally validated glossary, a second online survey was designed and launched to validate the glossary in the international community of WRD developers. The intended sample size for this study was set at 91 respondents, determined based on an estimated total target population size of N = 1000, a 95% confidence interval and 10% accepted margin of error [16, 17]. The full set of UA was divided into four batches so that respondents rated at least one of the batches. The division of the set was done to reduce the time required to complete the survey to under 15 min, aimed at increasing the completion rate. The UA in each batch were strategically distributed to balance the ones that had lower agreement scores from the local validation. The survey contained initial questions on demographics, and respondent’s experience in device development and usability evaluation, followed by the selection of one of the batches to rate (a) the respondent’s agreement with the proposed definition for each UA, (b) the relevance of the UA for the development of WRD and (c) the inclusion of the UA as a design criterion in the developments that the respondent was involved in. For all the ratings, a 5-point Likert scale was used. If the agreement rate for any UA definition was below 3, a text box was displayed giving the option to describe how they would improve or change the proposed definition. At the end of the survey, respondents could write down further comments in a text box and they could also choose to complete other attribute batches. The survey was reviewed and tested by four researchers with three different native languages (all proficient in the English language) to guarantee the understandability of the questions and face validity of the survey. The complete survey is available in Additional file 1: Annex 1.

All surveys were administered using the QuestionPro Survey Software (QuestionPro Inc., Austin, TX, USA). On the landing page of each survey, the study aims were presented, and informed consent was collected from the participants. Once the participants agreed with the stated terms and conditions, the surveys started. Data were collected from August 2022 to February 2023.

### Sample

The participants for the local validation were recruited through purposive and convenience sampling techniques, to guarantee valuable knowledge on the aspects studied and to allow performing on-site activities like the focus group in a timely manner, since they all were familiar with the IUT beforehand. An email was sent to the experts explaining the aim of the study, both the online survey and the focus group, and inviting them to participate in both or at least in the online survey. Inclusion criteria included experience in the development and usability evaluation of WRD, previous knowledge of the IUT, and a legally valid signature of the informed consent.

For the global validation stage purposive and snowball sampling techniques were used to obtain survey responses. Recruitment was made from the authors’ wider network via email, social media, the IUT website, and as well as at international conferences related to the field of WRD. Developers contacted through these channels were encouraged to take part in the survey emphasizing the importance of reaching a consensus regarding the definitions of usability attributes within the field. Their participation was underscored as vital for the validation of the glossary, ensuring a diverse range of respondents contributed to the process. Inclusion criteria included an agreement to participate in the survey and share the results (obtained at the beginning of the survey), and experience in the development and usability evaluation of WRD, assessed through four questions regarding this matter in the questionnaire. Additionally, there was a highlighted note in the introduction of the survey indicating that only WRD developers should complete it.

### Data analysis

All demographic variables and ratings are presented using descriptive statistics, either with their mean and standard deviation (mean ± STD) or with their median and quartiles first and third, Mdn (Q1–Q3), in case of high data dispersion. Categorical variables are analyzed with absolute frequency. Kolmogórov-Smirnov (KS) tests were performed for each demographic variable and rating to test for normal distribution. To further investigate whether professional experience influences the agreement, relevance or previous implementation of the UA included in the RUG, Spearman rank correlation tests were performed to assess possible correlations between each of the three ratings asked in the surveys and the professional data collected from the subjects: (i) years of experience as a developer, (ii) highest TRL achieved, (iii) the number of dedicated usability studies performed, and (iv) number of users they had previously interacted with. Lastly, the kurtosis and Pearson’s 2nd coefficient of skewness were calculated to study the distribution of the three ratings evaluated.

## Results

**Fig. 2:**
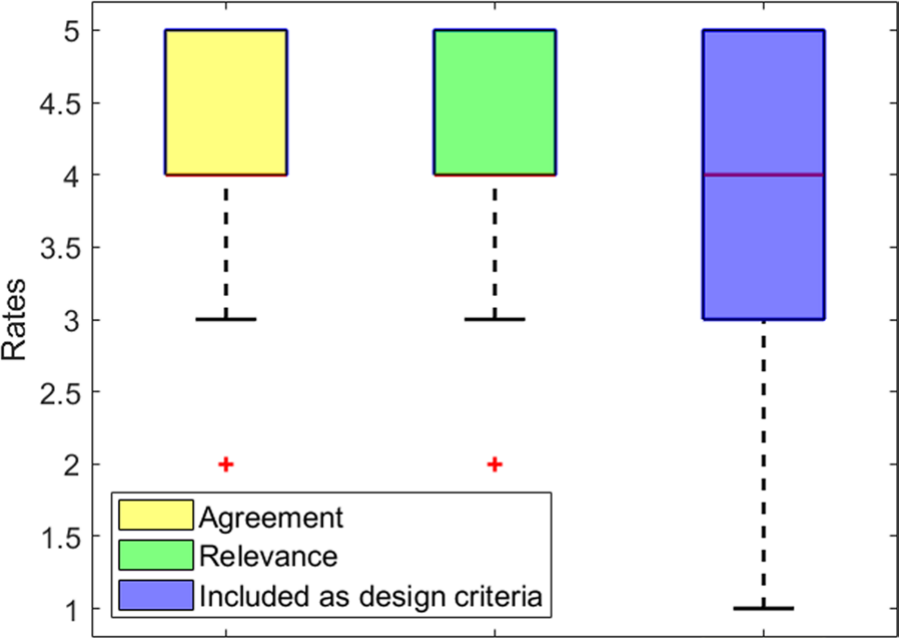
Box plots for each one of the three ratings assessed in the global validation stages for all the attributes

**Fig. 3:**
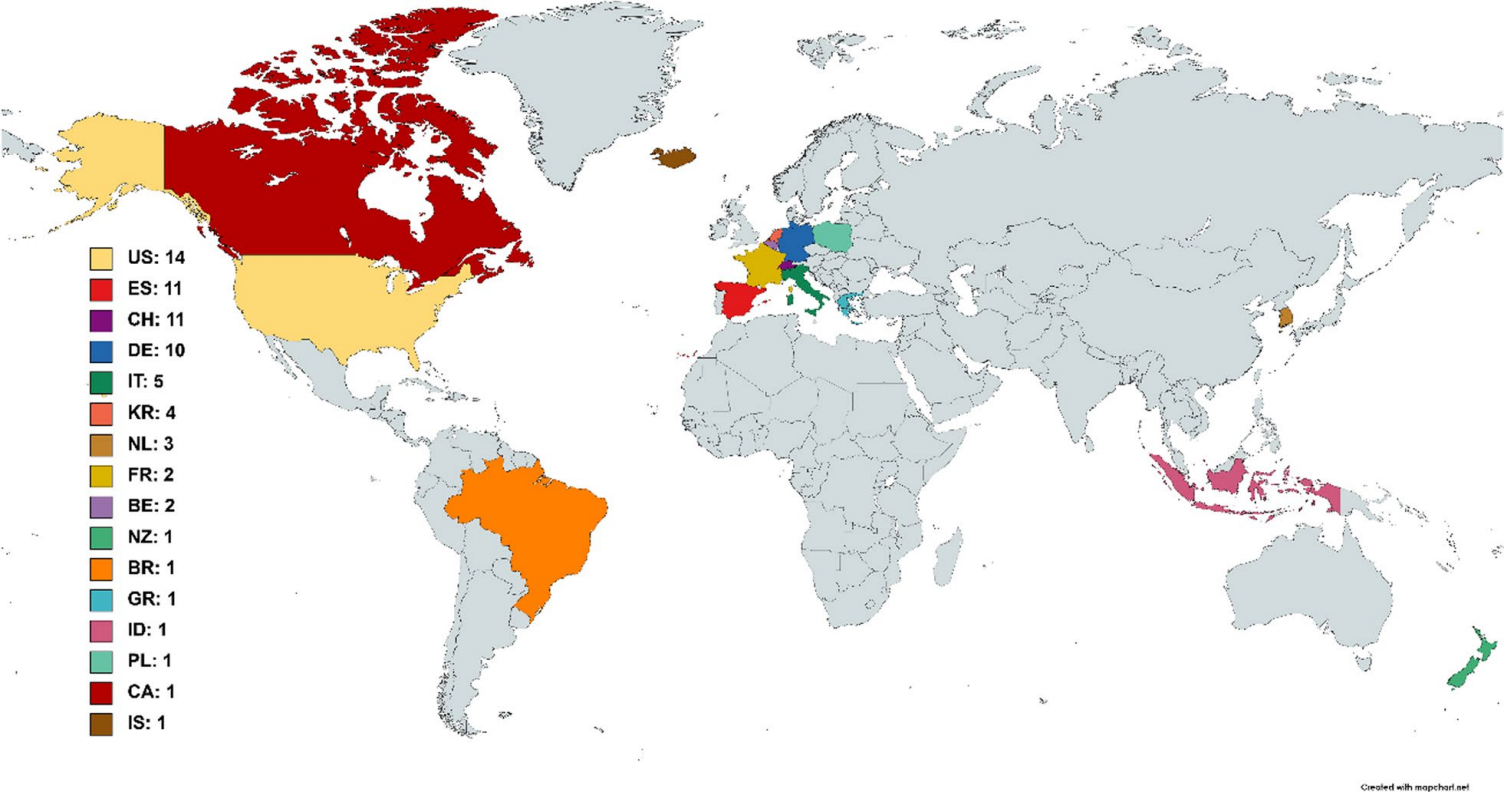
Respondents per country of the global validation stage. The acronyms used are United States (US), Spain (ES), Switzerland (CH), Germany (DE), Italy (IT), Korea (KR), Netherlands (NL), France (FR), Belgium (BE), India (IN), New Zealand (NZ), Brazil (BR), Greece (GR), Indonesia (ID), Poland (PL), Canada (CA) and Iceland (IS)

The local validation was performed with 7 WRD experts from ETH Zurich. In the global validation, 70 respondents from 17 countries around the globe participated. The participants' demographics and WRD experiences are summarized in Table 1. Only 20 UA were assessed during the local validation, since those were the ones for which the study coordinators (DHV, JTM) did not reach a consensus. Of these, only the 10 attributes that were not rated with an average agreement score of at least 4.0 were further discussed during the focus group. The participants of the focus group agreed on merging three out of five pairs of UA with similar definitions, preserving only the attribute that best encompassed both definitions. Therefore, by the end of the local validation, the glossary contained 43 UA to be externally validated. The list of the individual UA, their definitions and the ratings obtained in the global validation are available in Table 2. The full individual ratings obtained in both local and global validation stages are additionally included in Additional file 2*:* Annex 2. A summary of these ratings is shown in Table 3. Box plots showing the distribution of each type of rate among the 43 attributes are shown in Fig. 2. The average response time for this survey was 2.74 (2.05–4.02) min for the introductory part and 6.85 (4.80–11.85) min for the UA batches. The survey reached 713 viewers worldwide, of whom 150 started the survey and 70 fully completed it (completion rate = 46.67%). The geographical distribution of the respondents of the globally distributed survey is displayed in Fig. 3.

**Table 1:** Demographics and experience in the development of WRD of the respondents involved in the local and global validation of the UA glossary

Characteristic	Data	Local (n = 7)	Global (n = 70)
Age	Mean ± STD	29.7 ± 5.3	38.0 ± 11.0
Sex	Female	3	16
	Male	4	53
	Other	0	1
Countries	Total (Total)	4	17
Years involved in the development of WRD	Median (Q1–Q3)	3.0 (2.3–7.0)	7.0 (3.0–10.0)
No. of dedicated usability evaluation studies	Median (Q1–Q3)	2.0 (1.0–7.0)	2.0 (1.0–3.75)
No. of target users personally interacted with	Median (Q1–Q3)	15.0 (7.0–25.0)	15.0 (5.0–50.0)
Maximum TRL achieved	1 Basic research	0	2
	2 Technology formulation	0	1
	3 Needs validation	0	2
	4 Small-scale prototype	3	7
	5 Large-scale prototype	1	11
	6 Prototype system	1	8
	7 Demonstration system	1	12
	8 Initial commercialization	0	2
	9 Full commercial application	1	17

**Table 2:** Usability attributes of the glossary with their proposed definitions and the average ± STD ratings obtained in the global validation

Attribute	Proposed definition in the global validation survey	Agreement with the definition	Relevance in the field	Included as design criteria
Accessibility	The quality of being easily obtainable or reachable	4,00 ± 0,82	3,71 ± 1,21	2,71 ± 1,45
Adaptability	The ability of a system to change in order to suit different conditions	3,88 ± 1,45	4,13 ± 1,02	3,31 ± 1,35
Aesthetics	The extent to which a system's design is (visually) pleasing to the user	4,20 ± 0,95	3,45 ± 1,10	2,95 ± 1,15
Autonomy**	The capability of achieving a set goal within a defined scope without human interventions while adapting to operational and environmental conditions	3,83 ± 0,92	3,56 ± 1,20	2,94 ± 1,47
Cognitive load	The amount of mental effort required to perform a task	4,06 ± 1,34	4,12 ± 0,78	3,06 ± 1,14
Comfort*º	The extent to which the use of a system does not induce pain, unnecessary constraint or unpleasant feelings	4,33 ± 0,9	4,75 ± 0,58	4,31 ± 1,01
Compatibility*	The ability to work with other specific systems or environments without problems or conflict	4,20 ± 0,86	4,00 ± 1,03	3,00 ± 1,46
Complexity	The amount of effort needed to describe or use a system	3,71 ± 1,10	4,00 ± 1,00	3,56 ± 1,15
Cost-effectiveness	The degree to which something is effective or productive in relation to its cost	4,00 ± 1,32	3,65 ± 1,06	3,29 ± 1,26
Customizability	The ability to be modified to suit a particular individual, task, or environment	4,44 ± 1,03	4,13 ± 1,02	3,87 ± 1,19
Desirability*	The degree of how much a product is wanted by a user	4,27 ± 0,88	3,50 ± 1,21	2,69 ± 1,35
Durability	The ability to withstand wear, pressure, or damage	4,07 ± 1,00	3,93 ± 1,16	3,53 ± 1,60
Ease of use	The degree to which using a system is free of unnecessary effort	3,63 ± 1,15	4,33 ± 0,90	4,13 ± 0,99
Effectiveness	The accuracy and completeness with which users achieve specified goals	4,13 ± 1,15	4,63 ± 0,50	4,44 ± 0,81
Efficiency	The resources used in relation to the results achieved	4,26 ± 0,99	4,35 ± 0,81	3,95 ± 1,19
Embodiment	The perception of a beign part of the body-image as a feeling of ownership and agency	4,13 ± 1,15	3,44 ± 0,96	2,50 ± 0,82
Ergonomics	The degree of, or design for kinematic compatibility in a human–robot interface	3,06 ± 1,53	4,35 ± 1,06	3,76 ± 1,30
Error recovery	The quality of a system to allow the user to exit from a situation that the user did not intend to be in	4,24 ± 0,83	3,94 ± 1,20	2,63 ± 1,15
Feasibility	The determination as to whether assigned tasks could be accomplished by using the given resources	4,18 ± 0,88	3,94 ± 1,09	3,53 ± 1,37
Frustration	The feeling of being upset or annoyed as a result of being unable to change or achieve something	4,35 ± 0,79	3,59 ± 1,12	2,71 ± 1,53
Functionality*º	The extent to which the range of functions offered by a system can be used to perform the intended tasks	4,12 ± 0,86	4,56 ± 0,70	4,28 ± 0,89
Health benefit	The positive effect on a person's health gained from a system	4,53 ± 1,01	4,13 ± 1,26	3,44 ± 1,50
Helpfulness**	The ability of providing useful assistance	3,87 ± 1,3	4,00 ± 1,46	3,56 ± 1,63
Independence**	The ability to perform an activity with no or little help from others	4,53 ± 0,72	4,28 ± 1,07	3,72 ± 1,53
Intuitiveness*º	The extent to which a system is easy and natural to use, learnable and understandable	4,15 ± 0,93	4,30 ± 0,80	3,84 ± 0,90
Learnability	The ease and speed with which the users get familiar with the use of a system and retain these skills and knowledge	4,27 ± 0,88	4,14 ± 0,53	3,79 ± 0,97
Meet user needs*	The extent to which a system fulfills the design criteria given by the target user group	3,75 ± 1,18	4,63 ± 1,02	4,38 ± 0,96
Mobility*º	The ability to move in one's environment with ease and without restriction	4,47 ± 0,72	4,44 ± 0,70	4,17 ± 0,71
Motivation	The desire, or enthusiasm to do something (e.g. use a system or complete a task)	4,25 ± 0,85	3,75 ± 0,91	3,11 ± 1,10
Performance	The level of success in completing a task	4,24 ± 1,03	4,41 ± 0,62	4,35 ± 0,79
Physical workload	The amount and intensity of physical activity required to complete a task	4,16 ± 1,01	3,70 ± 1,08	3,60 ± 1,05
Pleasure*	A feeling of enjoyment or satisfaction, or something that produces this feeling	4,00 ± 0,69	2,94 ± 1,30	2,00 ± 1,08
Practicality*	The extent to being suitable for a particular occasion or use, or of being able to provide effective solutions to problems	3,61 ± 1,20	3,82 ± 0,88	3,41 ± 1,46
Quality of life	An individual's perception of their position in life in the context of the culture and value systems in which they live and in relation to their goals, expectations, standards and concerns	4,25 ± 1,07	4,10 ± 0,85	3,15 ± 1,14
Reliability*º	The extent to which a system will consistently perform its intended function adequately over time in a specific context of use	4,40 ± 0,75	4,70 ± 0,73	4,05 ± 1,00
Robustness	​The quality of being strong and unlikely to break or fail	3,95 ± 1,32	4,25 ± 0,97	3,95 ± 0,91
Safety*º	The extent to which the use of a system is free from danger or risk of injury	4,53 ± 0,51	4,76 ± 0,44	4,53 ± 1,01
Satisfaction	The extent to which the user's physical, cognitive and emotional responses that result from the use of a system meet the user’s needs and expectations	4,16 ± 1,01	4,50 ± 0,69	3,80 ± 1,06
Technical requirements*º	The set of technical design criteria required to deliver a desired function or behavior from a system to satisfy a user’s standards and needs	3,50 ± 1,51	3,13 ± 1,85	3,44 ± 1,50
Temporal demand	The amount of time required to complete a specific task using the system or in setting it up to be used	4,26 ± 0,99	4,21 ± 0,92	3,55 ± 1,05
Understandability**	The extent to which a system's functions and provided information are comprehensible	4,15 ± 0,99	3,79 ± 1,08	3,37 ± 1,30
Usefulness*º	The extent to which a system is effective in helping the user to do or achieve something in a practical way	4,47 ± 0,62	4,76 ± 0,56	4,24 ± 1,03
Wearability*º	The extent to which a WR can be mounted on the body and used without unnecessary movement restriction	3,88 ± 1,31 3,94 ± 0,97	4,69 ± 0,60 4,59 ± 0,87	4,13 ± 0,96 4,12 ± 1,17

The * indicates the attributes that were evaluated with the survey of the local validation stage whereas the ones marked with º indicate those that were discussed within the focus group. Attributes marked with ** were initially considered for merging due to similarities in their definitions with other UA in the set. However, they were not merged as per the focus group's decision

**Table 3:** Number of usability attributes of the glossary within a given range of ratings for each of the three questions included in the global survey. Specific attributes are shown for the lower scores

Rate	≥ 4.0	[3.5–4.0)	[3.0–3.5)	< 3.0
Agreement	32 UA	10 UA	1 UA (Ergonomics)	0 UA
Relevance	27 UA	12 UA	3 UA (Aesthetics > Embodyment > Technical Requirements)	1 UA (Pleasure)
Incl. Design	11 UA	14 UA	10 UA	8 UA

The thresholds stated in this table will be hereafter referred as the following categories: high rates ≥ 4.0, moderate rates [3.5–4.0), low rates [3.0–3.5) and very low rates < 3.0

KS tests indicated neither the demographic data nor the ratings followed a normal distribution, as can be confirmed with the skewness and kurtosis values. Poor Spearman rank correlations (|ρ|< 0.3) [18] were found between all the ratings and professional data from the respondents. These values are presented in Table 4.

**Table 4:** Spearman rank correlations for all three ratings and professional experience variables

Coefficient (ρ)	Agreement	Relevance	Incl. design
Years of experience	− 0.20	− 0.05	− 0.02
Max. TRL	− 0.11	0.03	0.14
Nº usability studies	− 0.10	0.11	0.27
Nº users interacted	− 0.17	0.04	0.18
Coefficient (ρ)	**Years exp**	**Max. TRL**	**Nº usability studies**
Years of experience	N/A	**0.51**	0.28
Max. TRL	**0.51**	N/A	**0.54**
Nº usability studies	0.28	**0.54**	N/A
Nº users interacted	0.34	**0.52**	**0.55**
Coefficient (ρ)	**Agreement**	**Relevance**	**Incl. design**
Agreement	N/A	0.26	0.16
Relevance	0.26	N/A	**0.62**

Correlation coefficients are considered very strong (|ρ|> 0.7), moderate (0.7 ≤|ρ|< 0.5), fair (0.5 ≤|ρ|≤ 0.3), or poor (|ρ|< 0.3) [18]. Moderate correlations are highlighted in bold

## Discussion

The objective of this work was to establish and validate a glossary of usability attributes aimed at improving usability evaluation practices to support the user-centered design of WRD. The established glossary, the RUG, provides a shared and validated terminology that is easily accessible and implementable by developers. To this end, our glossary facilitates the search and selection of context-specific outcome measures and usability research methods within the online Interactive Usability Toolbox (IUT) of ETH Zurich [14]. The generalizability and validity of the UA definitions comprised in our glossary were supported by the ratings of 70 developers of WRD from 17 countries around the world, who showed high agreement (≥ 4.0) on 32 of the 43 UA, and moderate agreement (4.0 > agreement ≥ 3.5) on other 10 UA. Likewise, developers agreed on the relevance of most of these attributes in the field of WRD, with 27 UA considered as highly relevant (≥ 4.0) and other 12 as moderately relevant (4.0 > relevance ≥ 3.5). Improved definitions for the attributes considered relevant but with moderate or low agreement ratings are also proposed based on the feedback provided by the respondents. All the comments provided by the respondents and the improved definitions are included in Additional file 2: Annex 2.

The high agreement ratings for most of the UA included in our glossary underline that, despite the wide interpretation of UA in the literature [6–9] our definitions are in general adequate and could serve as reference for future studies or for people interested in comprehensive usability evaluation of WRD. It is interesting to highlight that most UA with moderate or high-to-moderate agreement ratings are terms usually found within the field of engineering, e.g. *autonomy*, *complexity*, *robustness*, *technical requirements* and *wearability* [11]. We hypothesize that most developers possess an engineering background, which may lead them to interpret these terms in alignment with engineering-based definitions. Consequently, when prompted to provide a perspective on these terms from a different field, such as usability, discrepancies may arise. Widening the perspective of research and development teams beyond the engineering requirements is fundamental to promote the development of WRD that are usable and effectively respond to users’ needs [2].

A special case is that of *ergonomics*, the only attribute with low agreement but with high relevance. *Ergonomics* is a very wide umbrella term used differently across different fields and, thus, can be understood in different ways. In fact, this was the attribute that received the most comments. Instead of considering it as part of usability, *ergonomics* has long been studied as a separate field of research interacting with usability [19] and there are longstanding international efforts such as the Ergonomics Research Society or the International Ergonomics Association [20], that have stated definitions of the term *ergonomics* that can be adapted to suit specific fields. Consequently, several of the aspects regarding *ergonomics* relate also to usability, including other UA of our glossary such as *comfort* or *wearability*, and therefore, some WRD developers might consider that the whole field of *ergonomics* cannot be synthesized as a single, specific UA. Due to its high relevance, we consider it crucial to integrate *ergonomics* into the IUT, enabling developers to access the available tools for assessing the *ergonomics* of WRD, even though simplifying the entire field as a UA may be an oversimplification. Based on the feedback provided by the respondents and the definitions stated by the aforementioned organizations, the improved definition for *ergonomics* in the RUG is “the degree to which the interactions among users and elements of a WRD are optimized to increase human well-being and overall system performance including anatomical, anthropometric, physiological and biomechanical characteristics that relate to the intended use of a WRD”.

Complementary to the high agreement ratings obtained, the high (27 out of 43) and moderate (12 out of 43) relevance ratings of most UA underscore the multifaceted nature of usability. This observation highlights that usability is not a singular, simplistic concept but rather a complex interplay of various dimensions and attributes [16]. Consequently, to conduct a comprehensive assessment of usability, it becomes evident that multiple attributes of usability must be taken into consideration, highlighting the necessity for a holistic evaluation approach that transcends the prevalent trend in the field. Currently, the field predominantly relies on the use of three dimensions to describe usability (i.e. *effectiveness*, *satisfaction*, and *efficiency*) and usability evaluation is predominantly related to functional or performance-related outcomes [21, 22], followed by the evaluation *ease of use*, *safety* and *comfort* [16, 23], which may overlook the richness of usability. As expected, in our survey, many of the most widespread attributes related to the usability of WRD received very high relevance ratings (≥ 4.5): *safety*, *usefulness*, *comfort*, *reliability*, *wearability*, *effectiveness*, *functionality*, *meet user needs*, and *satisfaction*. However, *efficiency* received a high but not very high rate, indicating that other attributes are more relevant to the developers than only the three stated by ISO 9241–11. The glossary provided within this study, which deems most UA as relevant, signifies that the UA summarized and validated therein serve as pivotal elements that effectively encapsulate and represent the entirety of usability. A detailed analysis of the individual ratings (see Additional file 2: Annex 2) raises the need to debate whether the four attributes with relevance scores below 3.5 should be included in the glossary. *Aesthetics* and *embodiment* have borderline low-to-moderate relevance. Since they have been previously found to be design criteria important for the primary users of WRD under comparable terms such as “appearance” and “avoid machine body disconnection” [2], respectively, we consider they should be included in the list of UA of the IUT. Both definitions stated for these UA have high agreement, therefore, they do not need improved descriptions but rather more awareness from developers to be included as part of their design criteria, because both have poor scores in this regard. On the other hand, the UA *technical requirements* received a low relevance score and exhibited borderline moderate-to-low agreement among respondents. Comments associated with this attribute suggest that developers do not necessarily perceive it as an integral component of usability but rather believe that *technical requirements* and usability requirements are complementary in technology developments. Considering this valuable feedback, it is prudent to consider removing this attribute from the glossary. On the other hand, *pleasure* stands as the only UA marked with a low relevance score, albeit displaying high agreement in its definition. A detailed examination of the definition provided for this UA shows that it could be closely intertwined with the attribute of *satisfaction*, which holds very high relevance in the field. Hence, it may be reasonable to also consider omitting *pleasure* from the set of UA. Both UA are closely related to two psychology-related codes expressed by end-users of lower limb robotic devices for gait rehabilitation, including “positive feeling of being able to stand up and walk again” and “sense of wellness (physical and/or mental)” [2], underlining their relevance for end-users.

From the remaining 41 attributes, improved definitions were proposed for eight UA considered highly relevant (≥ 4.0) but with moderate (*adaptability*, *complexity*, *ease of use*, *helpfulness*, *meet user needs*, *robustness*, and *wearability*) or low (*ergonomics*) agreement ratings. In fact, most of these UA were the ones that more respondents commented on: *ergonomics* (10 comments), *adaptability*, *helpfulness*, *wearability*, and *technical requirements* with 4 comments each, and *robustness* and *durability* with 3 comments each. Three of these attributes (*ease of use*, *meet user needs*, and *wearability*) are also often included as design criteria (ratings ≥ 4.0), underpinning the importance of providing definitions that are agreed upon by developers in the field.

Moreover, a detailed analysis of the boxplots in Fig. 2 and the summary of the ratings in Table 3, show that while most of the attributes of the glossary are considered relevant in the field of WRD and that there is a high agreement with their proposed definitions, they have not been often included as design criteria in previous developments [16]. This can be confirmed by comparing the respondents’ years of experience in the field (mdn = 7) and the number of dedicated usability studies performed (mdn = 2). Therefore, our study underlines that usability is still poorly considered as part of the design criteria during device development, even if developers recognize its relevance. Actually, 10 respondents (17.14%) indicated that they had not performed any dedicated usability study in their career and two respondents (2.86%) reported they had never had contact with end-users of their devices. We consider there must be a paradigm shift in WRD development towards implementing user-centered design to properly address users’ needs during device developments [24–26], since it is unlikely that developments done without both involving users [27] and considering usability issues will be successful in reaching end-users [1, 28, 29].

It is worth noting that the highest correlation among all the studied combinations was found between the ratings of “relevance in the field” and “previously included as design criteria in technology developments” (moderate correlation, ρ = 0.62, p-value ≈ 0.00). This could be explained by the fact that developers may only include as design criteria the attributes that they consider relevant and overlook the ones that they do not consider important. In fact, the eight UA seldomly included as design criteria (ratings < 3.00) are not considered highly relevant in the field (relevance < 4.0). These are *accessibility*, *aesthetics*, *autonomy*, *desirability*, *embodiment*, *error recovery*, *frustration*, and *pleasure*. All of these UA exhibit high or moderate (only in the case of *autonomy*) agreement in their respective definitions. Therefore, their infrequent inclusion as design criteria, despite their moderate relevance scores, cannot be attributed to having ambiguous definitions. Instead, this pattern illustrates that some UA are potentially less relevant in specific application cases of WRD or could arise from a potential lack of awareness regarding their significance from the perspective of end-users. It's important to note that all the listed UA originally emerged as design criteria demanded by primary or secondary end-users in a prior study on lower limb WRD [2].

A moderate correlation between the professional experience related to the “number of dedicated usability studies performed” and the “number of users personally interacted” was found (ρ = 0.55, p-value ≈ 0.00). This can be easily understood because the more usability studies performed, the more users are involved in these studies. Similarly, more users must be involved in usability evaluation as technology becomes more mature, which explains the positive correlation between higher TRLs and both the “number of usability studies performed” (ρ = 0.54, p-value ≈ 0.00) and “number of users personally interacted” (ρ = 0.52, p-value ≈ 0.00). In this regard, results show that the peak values for both user involvement and usability studies are in late TRLs (i.e. 6, 8 and 9), corresponding to the stages of prototypes validated and product. Similar results were found in a previous study [16], highlighting the relevance of user involvement to develop technologies that go beyond the prototype phase and successfully reach end-users [30].

Previous efforts to define usability in WRD [7, 8] contained 17 attributes each and agreed on seven of them. Nonetheless, some of them are related to services that must be provided by the distributors of the WRD or are entirely device-centered. Moreover, in contrast to our work, none of these models validated the attributes and their definition within the local or global community of WRD developers, limiting the diffusion, impact, and generalizability of the proposed glossaries. Therefore, their selection of terms for what is considered usability was arbitrary, and some of the proposed definitions are not specifically related to usability. The RUG comprises all the UA included in previous efforts and provides definitions specifically related to usability, including the four UA included in the COST action dictionary and the factors and subfactors in the EXPERIENCE questionnaire from Eurobench [11, 12]. The detailed comparison between these previous works in the field and the attributes of our glossary that encompass their definitions are presented in Additional file 3: Annex 3.

Therefore, the RUG is the most comprehensive set of UA available in the field of WRD to evaluate usability and has been externally assessed and improved by developers from most of the active countries working in the field of WRD, thus enhancing its generalizability. It can be readily accessed through the IUT website (www.usabilitytoolbox.ch), enabling developers to have immediate open access to the definitions of each UA and to identify context-specific outcome measures and usability evaluation methods related to each attribute. Three examples are presented in Table 5. The results of this study do not aim to point to specific attributes as being more important than others, but rather underline that all attributes should ideally be considered for a holistic usability evaluation. Despite the glossary being built entirely in English, it was mostly agreed upon by both native and non-native English speakers. In fact, all the definitions within our glossary are not aimed exclusively at the field of WRD but were rather built from a usability perspective. This means that they could possibly be useful to be implemented in other fields related to wearables, robotics, and health technologies overall. In case such interest arises, we recommend engaging developers from each specialized field to evaluate the significance of the attributes included in our glossary and the appropriateness of the proposed definitions within their respective domains. This evaluation is advised before directly implementing the current glossary.

**Table 5:** Examples of measurement tools selected using the IUT to evaluate specific usability attributes of three different WRD for different target users: an upper limb WRD for amputated children, an augmentation lower limb WRD for adults, and a lower limb WRD for gait rehabilitation of post-stroke adults

Upper limb WRD for amputated children	Augmentation lower limb WRD for adults	Lower limb WRD for stroke therapy of adult population
Comfort	Effectiveness	Effectiveness
Assistive Technology Device Predisposition Assessment (ATD-PA) Michigan Hand Outcomes Questionnaire Product Emotion Measurement Tool Visual Analog Scale Evaluation grid Numeric Rating Scale	Kinetic and kinematic analysis Physiological measures Task analysis	Kinetic and kinematic analysis Physiological measures Task analysis Fugl-Meyer Assessment Foot and Ankle Ability Measures Mini Balance Evaluation Systems Test Short Physical Performance Battery
Durability	Efficiency	Efficiency
Visual Analogue Scale 3D models and simulations Evaluation grid Failure Mode and Effect Analysis	Subjective Workload Assessment Technique Time for Task Task Analysis Perlman’s Practical Heuristics for Usability Evaluation	Subjective Workload Assessment Technique Time for Task Task Analysis Perlman’s Practical Heuristics for Usability Evaluation
Functionality	Satisfaction	Satisfaction
Canadian Occupational Performance Measure Children Amputee Prosthetics Projects—Prosthesis Satisfaction Inventory Assistive Technology Device Predisposition Assessment Upper Extremity Function Test Action Research Arm Test Physiological measures Kinetic and/or kinematic analysis Evaluation grid Accessible Usability Scale Box and block test Hand-held dynamometer	System Usability Scale Self-Assessment Manikin Net Promoter Score	System Usability Scale Self-Assessment Manikin Net Promoter Score Short Form-36 Health Survey Questionnaire Utrecht Scale for Evaluation of Rehabilitation–Participation Stroke Specific Quality of Life Scale

### Limitations and future work

The estimated target sample size of the global validation stage was not fully met. Nevertheless, in line with the previous online survey experience of the research team [16], all measures to reach the largest possible sample were taken. The survey was widely shared through several channels (e.g. social media, conferences, email lists, research centers and companies, the IUT website, and Exoskeleton Report) to reach WRD developers from different countries and from both academia and industry. Additionally, the data collection period was extended until there was no increase in the responses gathered. To increase the completion rate, the survey was designed dividing the glossary into the UA batches to guarantee a reasonable response time (below 10 min.). Nevertheless, this raises an additional limitation to the study, since not all respondents rated all UA, representing a possible confound. The authors gave priority to increasing the number of responses collected, since the main objective of the study was to obtain an external validation of the glossary with the participation of a wide sample of respondents.

Collecting the professional background information of the respondents in the global survey would have enabled us to explore potential correlations between each rating and the respondents' profiles. This is important because some respondents may have a technical development-oriented perspective, while others might have professional backgrounds more closely aligned with being end-users of the technologies (e.g. clinicians or people with neurological injuries), thereby reflecting perspectives from real-life scenarios. The current study purposely targeted only technology developers because they are mostly the ones conducting and designing usability evaluations or WRD. Therefore, we aimed to reach a consensus among them. Nevertheless, understanding that there might be differences between end-users and developers regarding the perception and relevance of the usability attributes, it would be interesting to perform another study targeting only end-users. The study would be aimed at comparing the understanding and relevance of the UA included in the RUG and to check if end-users identify additional usability attributes that ought to be added to the glossary. Such an effort would require a different survey and different distribution channels to the ones used in this work. We strongly suggest including a question to identify the background of the respondents in the survey and assess possible differences in their responses. As indicated before, this is an important limitation of our study.

Another limitation of our effort is that the proposed methodology was aimed at reaching an external validation of the glossary but could instead be considered a participative assessment and improvement of the proposed definitions. Therefore, it remains as a somewhat subjective methodology, because we did not implement our global validation stage as a truly iterative process with multiple rounds of evaluation where participants could reach a consensus. Ideally, the global validation could have taken the form of an e-Delphi study [31], but such an approach is highly resource and effort demanding, which might have further limited the participation of developers. We consider that the participation of developers from several countries and with different native languages was fundamental to making the glossary generalizable, understandable, and representative to developers from all continents. For developers interested in translating the RUG to other languages, we strongly suggest such translation is performed carefully by native speakers with knowledge of the field, to make sure the specificity of the terms is preserved. Lastly, it might be worth to regularly updating the RUG based on the potential emergence of new disruptive technologies, because WRD is still a developing field. Doing it is important to assess if new attributes are needed when such devices appear in the field. A new survey can be carried out to this end. If performed, we strongly suggest also considering the application(s) of the WRD with whom respondents have experience. This is important because the relevance of certain usability attributes can depend on the application of a given WRD, as it already discussed in our paper. Alternatively, any other type of global coordinated effort between leading organizations in the field or WRD can lead to an updated version of the RUG when considered necessary by the demands of the people working in the field.

## Conclusions

Our glossary provides a comprehensive set of UA in the field of WRD to evaluate usability. The generalizability and relevance of these UA were supported by the ratings of 70 developers of WRD from 17 countries around the world. These results signify that the UA summarized and validated in our glossary serve as pivotal elements that effectively encapsulate and represent the entirety of usability. To conduct a comprehensive assessment of usability, multiple attributes of usability must be taken into consideration, in contrast to the prevalent trend in the field. Our study underlines that usability is still poorly considered part of the design criteria during device development, even if developers recognize its relevance. In this regard, there seems to be a lack of awareness regarding the significance from the perspective of end-users of some UA considered moderately relevant but seldom included during device development.

Overall, this effort is aimed at improving usability evaluation practices during the development of WRD by providing a shared and validated terminology that is easily accessible and implementable by developers, and that can lead to the definition of benchmarks for usability evaluation to promote the acceptance of WRD.

### Supplementary Information

**Additional file 1.** Template of the global survey used to rate the agreement, relevance and recorded use of the usability attributes included in the glossary.

**Additional file 2.** Definitions of each usability attribute and their individual ratings obtained in both local and global validation stages.

**Additional file 3.** Detailed comparison between the other usability models available in the field and the usability attributes included in the RUG.

## Data Availability

All new data created in this study is provided in the manuscript and the three Additional files provided.
