# Supplementary material for: Towards a validated glossary of usability attributes for the evaluation of wearable robotic devices
Source: J Neuroeng Rehabil. 2024 Feb 28;21:30. doi: 10.1186/s12984-024-01312-1 (PMC10900611; doi:10.1186/s12984-024-01312-1)
Supplement: Supplementary file 1 — Additional file 1. Template of the global survey used to rate the agreement, relevance and recorded use of the usability attributes included in the glossary. [file 12984_2024_1312_MOESM1_ESM.pdf]

Dear participant,

You are invited to participate in our survey to reach a consensus on the definitions for attributes commonly used to describe the usability of wearable robotic devices (WRD). These attributes are the basis of usability evaluation protocols for WRD and will allow the community to find and create more widely accepted benchmarks. These efforts are linked to the WRD usability glossary provided by the Interactive Usability Toolbox (www.usabilitytoolbox.ch) of ETH Zurich. **Please complete this survey only if you have developed and tested wearable robots.**

In this survey, **we ask you to rate a set of definitions and their applicability to the development of wearable robotic devices**. It will take approximately **15 minutes to complete** the survey. We would ask you to complete at least one set of selected usability attributes (all the attributes were divided in four batches).

The data will be stored anonymously on the secured servers of QuestionPro (temporary) and ETH Zurich (permanently), with access limited to the survey coordinators. The anonymized data may be shared and made public. You are free to stop the survey at any time.

By clicking "Start" below, you agree to these terms and conditions.

For further questions, contact us directly via mail at: dianasofia.herreravalenzuela@hest.ethz.ch

Thank you very much for your time and support.

1) Please specify your age (in years)
2) What is your sex?

   M, F, Other

3) How many years have you been actively involved in research and/or development of wearable robotics devices?
4) Please indicate the furthest Technology Readiness Level (TRL, 1 to 9) which you have reached with a wearable robotic device. A level is achieved only when the description of the level is completed (see below).

   Please only refer to work where you have been actively involved. (TRL Chart)

   Slider 1-9

5) Over the course of your career, **how many dedicated usability evaluation studies** with wearable robotic devices have you performed (approximately)?
6) With how many **target users** have you *personally* tested your device(s), or interacted during the design process of your device(s)? An approximation or estimate is fine.

Now we will start with the ratings of the usability attributes. If you have any comments or extended thoughts, please take note of them. At the end of the survey, you have an open feedback section where you can write them down. Any detail would be very useful for us.

7) Please choose one of the usability attribute batches indicated below. Each batch contains 11 attributes from our full list of 44 usability attributes. If you want to rate more than one batch, at the end of the survey you will have the opportunity to complete additional ones. More responses will allow us to have a broader validation of the definitions.

A, B, C, D

E.G. Usability Attributes Batch A

Thank you for choosing to complete a batch of usability attributes. For the following questions, please indicate your agreement with the definitions or the attribute relevance with a star rating from 1 (lowest) to 5 (highest).

1. Please rate your agreement for the following definition of Autonomy as a usability attribute:

	1	2	3	4	5
The capability of achieving a set goal within a defined scope without human interventions while adapting to operational and environmental conditions.	❏	❏	❏	❏	❏

How would you improve or change the definition? [Optional] Autonomy: The capability of achieving a set goal within a defined scope without human interventions while adapting to operational and environmental conditions.

How relevant is Autonomy (understood as the previous usability-related definition) as a usability attribute for the development of wearable robotics?

	1	2	3	4	5
Relevance	❑	❑	❑	❑	❑

In the development of the wearable robots you have participated, how often have you included Autonomy (understood as the previous usability-related definition) as a design criteria?

	1	2	3	4	5
Frequency	❑	❑	❑	❑	❑

<u>(11 Attributes with the same format in each batch)</u>

Do you have any comments or suggestions regarding the Usability attributes comprised in your survey?

Do you want to rate another batch of usability attributes? More responses will allow us to have a broader validation of the definitions.

1. Yes, Batch A
2. Yes, Batch B
3. Yes, Batch C
4. No, I am done
